# A partitioned 88-loci psoriasis genetic risk score reveals HLA and non-HLA contributions to clinical phenotypes in a Newfoundland psoriasis cohort

**DOI:** 10.3389/fgene.2023.1141010

**Published:** 2023-05-31

**Authors:** Audrey Bui, Sugandh Kumar, Jared Liu, Faye Orcales, Susanne Gulliver, Lam C. Tsoi, Wayne Gulliver, Wilson Liao

**Affiliations:** ^1^ Department of Dermatology, University of California San Francisco, San Francisco, CA, United States; ^2^ Lake Erie College of Osteopathic Medicine, Bradenton, FL, United States; ^3^ NewLab Clinical Research Inc, St. John’s, NL, Canada; ^4^ Department of Dermatology, University of Michigan, Ann Arbor, MI, United States; ^5^ Department of Computational Medicine and Bioinformatics, University of Michigan, Ann Arbor, MI, United States; ^6^ Department of Biostatistics, University of Michigan, Ann Arbor, MI, United States; ^7^ Faculty of Medicine, Memorial University of Newfoundland, St. John’s, NL, Canada

**Keywords:** genetic risk score (GRS), polygenic risk score (PRS), psoriasis, Newfoundland and labrador, genetics, HLA

## Abstract

Psoriasis is an immune-mediated inflammatory skin disease typically characterized by erythematous and scaly plaques. It affects 3% of the Newfoundland population while only affecting 1.7% of the general Canadian population. Recent genome-wide association studies (GWAS) in psoriasis have identified more than 63 genetic susceptibility loci that individually have modest effects. Prior studies have shown that a genetic risk score (GRS) combining multiple loci can improve psoriasis disease prediction. However, these prior GRS studies have not fully explored the association of GRS with patient clinical characteristics. In this study, we calculated three types of GRS: one using all known GWAS SNPs (GRS-ALL), one using a subset of SNPs from the HLA region (GRS-HLA), and the last using non-HLA SNPs (GRS-noHLA). We examined the relationship between these GRS and a number of psoriasis features within a well characterized Newfoundland psoriasis cohort. We found that both GRS-ALL and GRS-HLA were significantly associated with early age of psoriasis onset, psoriasis severity, first presentation of psoriasis at the elbow or knee, and the total number of body locations affected, while only GRS-ALL was associated with a positive family history of psoriasis. GRS-noHLA was uniquely associated with genital psoriasis. These findings clarify the relationship of the HLA and non-HLA components of GRS with important clinical features of psoriasis.

## 1 Introduction

Psoriasis is an immune-mediated chronic inflammatory disease characterized by erythematous and scaly skin plaques. It affects 3% of the Newfoundland population while only affecting 1.7% of the general Canadian population (Nall et al., 1999; Papp et al., 2011). The Newfoundland province of Canada is a genetically isolated population comprising approximately 500,000 residents with 98% English or Irish descent (Rahman et al., 2003). Because Newfoundland rose from a limited founder population, it is an exceptional resource for studying familial disorders such as psoriasis which was found at a higher prevalence in Newfoundland compared to other white populations (Nall et al., 1999).

Although environmental triggers like stress, infection, and trauma can contribute to the development of psoriasis, family-based and population studies suggest an important genetic component to the development of psoriasis (Alshobaili et al., 2010; Chandran and Raychaudhuri, 2010; Capon, 2017). The pathogenesis of psoriasis involves the dysregulation of T cells, antigen presenting cells, and keratinocytes among many cell types. Genome-wide association studies (GWAS) in psoriasis have identified many susceptibility loci with modest individual effects (Cargill et al., 2007; Stuart et al., 2015; Tsoi et al., 2017). Among these, the HLA-C*06:02 allele is thought to contribute the greatest genetic effect (Henseler and Christophers, 1985). A prior study showed that with each additional HLA-C*06:02 risk allele (tagged by rs10484554), there was a 206% elevated risk of psoriasis (Chen et al., 2011). However, HLA-C*06:02 only accounted for 6.7% of the genetic heritability found in psoriasis, suggesting the importance of other HLA genes and non-HLA genes in disease progression (Chen et al., 2011).

While psoriasis commonly presents on the scalp and extremities, it often first occurs on the extensor surfaces such as the elbows and knees. Psoriasis is classified into two types: Type 1 psoriasis (T1P) which starts before age 40, and T2P which starts at or after the age of 40 (Henseler and Christophers, 1985). T1P has been associated with a family history of psoriasis with involvement of HLA-C*06:02 whereas T2P is less associated with a positive family history or involvement of HLA-C*06:02.

Prior studies have shown that combining many genetic susceptibility loci into an overall genetic risk score (GRS) can improve identification of people at risk for that disease (Weedon et al., 2006; Meigs et al., 2008; Abraham et al., 2021). Previous GRS studies in psoriasis have shown that GRS can be used for psoriasis disease prediction and shows an inverse correlation with age of onset (Chen et al., 2011; Lu et al., 2013; Tsoi et al., 2017). However, there is a lack of studies investigating the association of GRS with psoriasis clinical features.

In this study, we calculated three types of GRS: one using all known GWAS SNPs (GRS-ALL), one using a subset of SNPs from the HLA region (GRS-HLA), and the last using non-HLA SNPs (GRS-noHLA). In past GWAS studies of psoriasis, *HLA-C* has been identified as a highly associated locus. Our lab and others have uncovered many functional roles for HLA genes, so it is intuitive to set these SNPs apart as a functionally separate group of SNPs as they play a major role in antigen presentation (Chen et al., 2012; Zeng et al., 2013; Yanovsky et al., 2020; Ahn et al., 2021). The partitioning of non-HLA SNPs was performed to investigate their possible role independently from the HLA SNPs. We examined the relationship between these GRS and several psoriasis features within a Newfoundland cohort. These include family history, age of onset, psoriasis severity, locations ever affected by psoriasis, locations first affected by psoriasis, and total number of locations. In addition, we explore how the association of GRS with these clinical features is modified by early-onset psoriasis *versus* late-onset psoriasis (T1P vs T2P).

## 2 Materials and methods

### 2.1 Cohort

The study cohort includes 654 psoriasis cases of European ancestry from the Newfoundland region of Canada. Psoriasis patients were residents of Newfoundland and Labrador that were clinically diagnosed with psoriasis by a dermatologist in the late 1980s and early 1990s. The cohort consisted of 52% female and 48% male patients with and average age of 41 with a standard deviation (SD) of 14. Clinical data collected included birthplace, maternal heritage, paternal heritage, race, BMI, gender, age of onset, relatives with psoriasis, body locations ever or first noted with psoriasis (hand, back of hand, palm, foot, sole, toe, nail, scalp, face, neck, arm, elbow, armpit, leg, knee, back, chest, trunk, genital area, other), other serious illnesses, psoriasis severity as rated by the dermatologist (i.e., mild, moderate, severe), and type of psoriasis (i.e., plaques, guttate, pustular, *etc.*). All data fields and category options are included in Supplementary Table S1. All subjects provided written informed consent for use of their data and biosamples under IRB approval HREB #2019.188.

The independent validation cohort data were collected from 345 psoriasis patients of European descent at the University of California San Francisco (UCSF) Department of Dermatology enrolled between 2006 and 2016. The diagnosis of psoriasis was confirmed by a board-certified dermatologist, and study subjects completed a survey including demographic characteristics, medical history, and clinical features. The cohort consisted of 48% female and 52% male patients with an average age of 49 and a SD of 16. All subjects provided written informed consent for use of their data and biosamples under UCSF IRB# 10-02830.

DNA from both cohorts was genotyped on the Affymetrix United Kingdom Biobank Axiom Array (ThermoFisher) using a GeneTitan Multi-Channel Instrument (Applied Biosystems). SNPs were called using Analysis Power Tools 2.10.2.2 (Affymetrix, https://www.affymetrix.com/support/developer/powertools/changelog/index.html). Sample and SNPs passed quality control using the parameters of call rate >97% and Dish QC >82%.The resulting genotype. vcfs were scanned with ‘snpflip’ (https://github.com/biocore-ntnu/snpflip) using the GRCh37 build of the human genome reference sequence maintained by the University of California, Santa Cruz (http://hgdownload.cse.ucsc.edu/goldenPath/hg19/bigZips/hg19.fa.gz) to identify reversed and ambiguous-stranded SNPs, which were flipped and removed (respectively) using Plink (http://pngu.mgh.harvard.edu/purcell/plink/) (Purcell et al., 2007), and the remaining sites were sorted using Plink (www.cog-genomics.org/plink/2.0/) (Chang et al., 2015). Clinical study data were managed using REDCap (Research Electronic Data Capture) hosted at University of California San Francisco (Harris et al., 2009; Harris et al., 2019). This web-based software provides an intuitive interface for validated data capture, audit trails for tracking data manipulation and exportation, automated export procedures, and procedures for data integration with external sources.

### 2.2 SNP selection

We constructed our GRS using 88 SNPs and their corresponding odds ratios (ORs) from the largest published psoriasis GWAS meta-analysis identifying SNPS meeting genome-wide significance (*p* < 5.0E-08) (Supplementary Table S2) (Tsoi et al., 2017). This meta-analysis included six GWAS, one exomechip, and one immunochip datasets of European ancestry. Many of the psoriasis loci from this study contained secondary independent signals. To ensure the effect sizes from the primary signals were not over- or underestimated, the ORs were calculated by conditioning on other independent signals within the same locus (Tsoi et al., 2017). This ensured the independent effect of each signal on psoriasis was properly represented despite any linkage disequilibrium (LD) structure. Moreover, because of the especially high LD with HLA loci, we calculated the pairwise linkage disequilibrium between the 11 HLA SNPs across a European population using the LDmatrix Tool within LDlink (Machiela and Chanock, 2015). This confirmed low LD between the 11 HLA SNPs selected, with 93% of the pairwise LD comparisons having an *R* ^2^ < 0.1.

### 2.3 Imputation

SNP data from the Affymetrix United Kingdom Biobank array were augmented with imputed SNPs from the Michigan Imputation Server (https://imputationserver.sph.umich.edu) (1000G Phase 3 v5 GRCh37 reference panel, rsqFilter off, Eagle v2.4 phasing, EUR population). SNP positions were translated to GRCh38 coordinates using the ‘LiftoverVcf’ command of Picard 2.23.3 (http://broadinstitute.github.io/picard/). The imputation quality of the 88 psoriasis SNPs was highly accurate with *R* ^2^ > 0.8 showing the high confidence and correct imputation of all SNPs (Supplementary Table S3). We utilized all 88 markers in our analysis as previous studies have indicated that utilizing imputation quality cutoffs has a detrimental impact on GRS discriminatory ability (Goldstein et al., 2015; Chen et al., 2020). Data is publicly available at https://doi.org/10.6084/m9.figshare.21970847.

### 2.4 GRS calculation

Initially, two approaches were used to calculate the GRS: the simple risk allele count (cGRS and the weighted method (GRS). Subsequent analyses found that the weighted GRS to be superior to cGRS, so only the weighted GRS results are presented. The weighted GRS was calculated as the sum of the number of risk alleles weighted by the OR of that allele. GRS calculation was performed on imputed continuous-valued dosages between 0 and 2 rather than number of risk alleles. The GRS-ALL was calculated as the weighted GRS using all 88 SNPs, the GRS-HLA was calculated using the 11 SNPs found in the HLA region (chr6:28510120-33480577 in GRCh38), and the GRS-noHLA was calculated using the 77 non-HLA SNPs. The calculations were performed in Plink.

### 2.5 Association testing

Association testing of GRS-ALL, GRS-HLA, and GRS-noHLA with psoriasis clinical features was performed using logistic regression or linear regression in R (v4.1.0). A *p*-value of less than 0.05 was deemed significant. The principal component analysis for genital psoriasis was performed in R (v4.1.0) using the prcomp () function in the stats (v4.1.0) package. The samples were grouped based on the presence of genital psoriasis. For each group, the top ten non-HLA SNPs in principal component 1 with the loadings of the greatest magnitude were further explored. Associations between the 88 loci and genital psoriasis were tested with SNPTEST v2.5.4 using the additive model (“-frequentist 1”) and genotype dosages imputed by MIS (“-method expected”) for all cohorts (Supplementary Table S4) (Wellcome Trust Case Control, 2007).

## 3 Results

### 3.1 Family history

**FIGURE 1:**
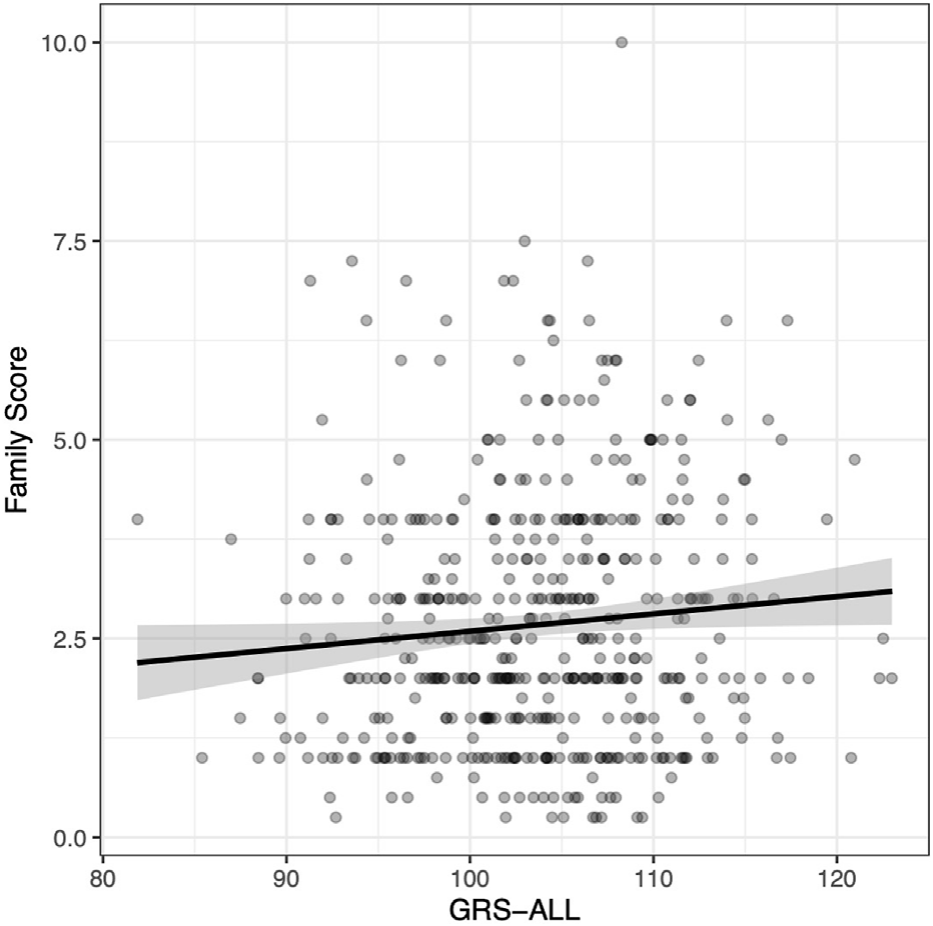
Positive correlation between family score and GRS-ALL. Coefficient = 0.022. *R* ^2^ = 0.0067, *p* = 0.038, SE = 0.011, n = 495. Family score calculated by summing the contributions from relatives affected by psoriasis as follows: first degree relative = 1, a second-degree relative = 0.5, a third-degree relative = 0.25.

The family score was calculated as the sum of all relatives diagnosed with psoriasis in which first-degree relatives were given a weight of 1, second-degree relatives were given a weight of 0.5, and third-degree relatives were given a weight of 0.25. A linear regression was used to evaluate the relationship between GRS and psoriasis family score. A significant positive association was found between GRS-ALL and the family score (*p* = 0.038; Figure 1) with and odds ratio (OR) [95% CI] of 1.02 [1.00-1.04]. Meanwhile, this association was found to be nonsignificant when using GRS-HLA and GRS-noHLA. These results indicate that all SNPs regardless of loci cumulatively contribute to the family history.

### 3.2 Age of psoriasis onset

**FIGURE 2:**
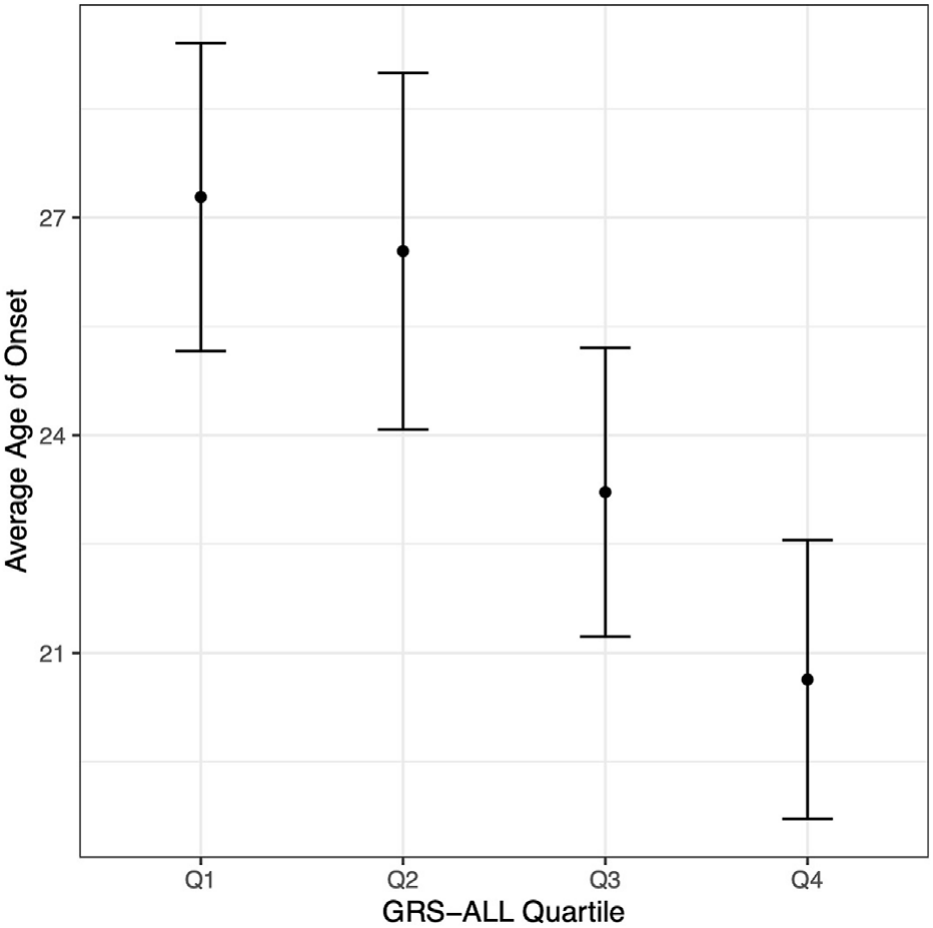
Age of onset decreases with increasing GRS-ALL. Point corresponds to average age of onset. Vertical bars correspond to 95% confidence intervals. n = 163 per quartile.

A linear regression analysis revealed a significant negative association between GRS-ALL and onset in which increasing GRS was associated with an earlier age of onset (coefficient = −0.341, standard error (SE) = 0.09, *R* ^2^ = 0.023, *p* = 0.0001, OR 0.71 [0.59-0.85]). The average age of onset was calculated for each GRS-ALL score quartile in which the first quartile had an average age of onset of 27.28 (SD = 13.76, SE = 1.07) while the fourth quartile had an average of 20.63 (SD = 12.43, SE = 0.97; Figure 2; Supplementary Table S5). The linear regression was repeated using GRS-HLA which yielded a more significant association (coefficient = −0.772, SE = 0.156, *R* ^2^ = 0.0398, *p* = 1.03 × 10^−6^, OR 0.46 [0.34-0.63]). Meanwhile, the results showed a similar trend but were nonsignificant when repeated with the GRS-noHLA. These results suggest that individuals with a higher GRS have an earlier age of onset and that this trend is driven by the HLA SNPs.

### 3.3 Psoriasis severity

**FIGURE 3:**
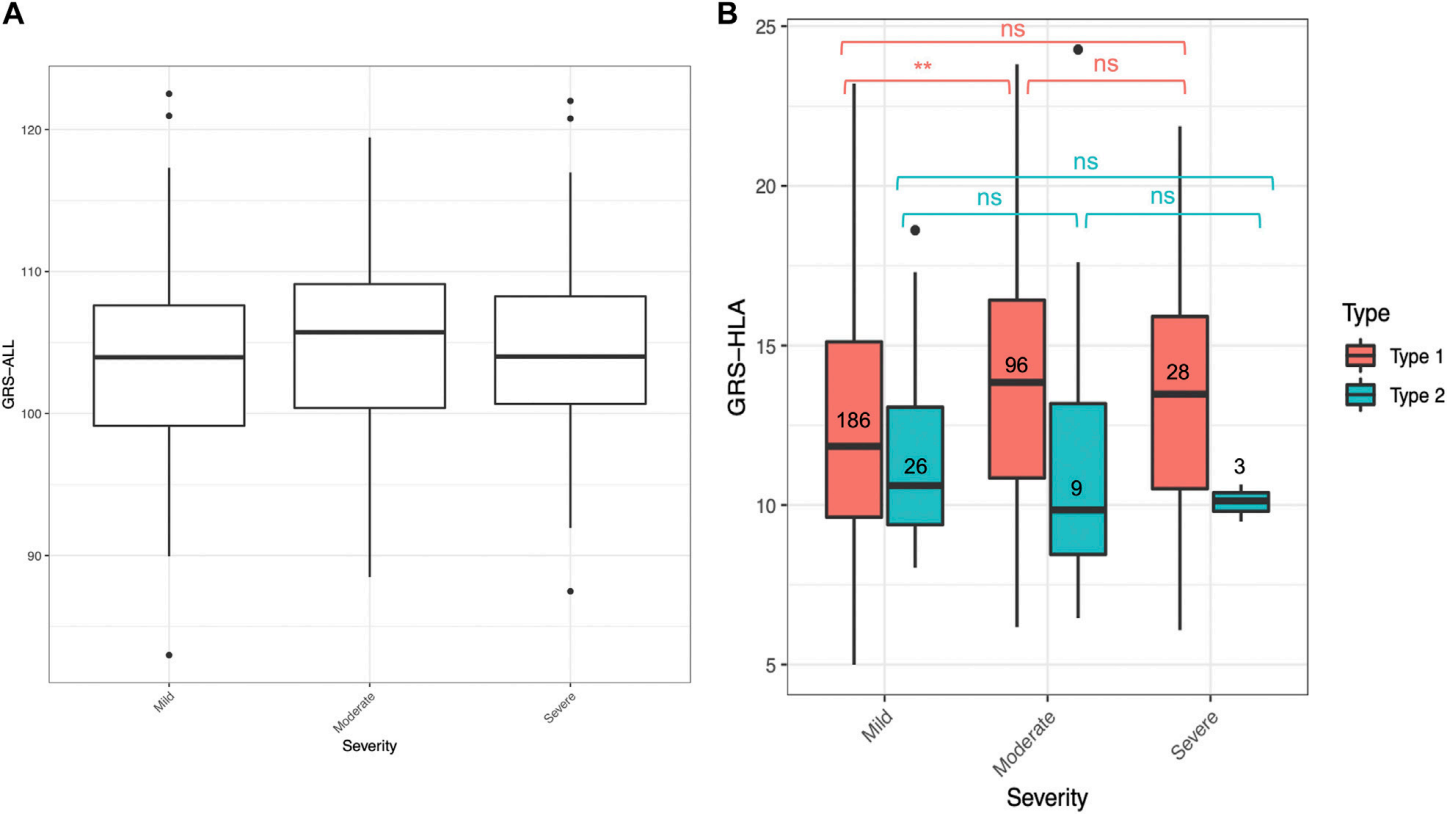
GRS for mild, moderate, and severe psoriasis. Horizontal lines represent quartiles. Points represent outliers. **(A)** Non-significant difference in GRS-ALL between severity levels when analyzed with Kruskal–Wallis test. n = 265 (mild), 112 (moderate), 32 (severe). **(B)** Significant difference in GRS-HLA between mild and moderate in Type 1 psoriasis. Kruskal–Wallis Test *p ≤ 0.05, **p ≤ 0.01, ***p ≤ 0.001.

An ordinal logistic regression was performed to evaluate the relationship between GRS and psoriasis severity level, categorized as mild, moderate, and severe, as rated by a dermatologist. The patients were categorized based on dermatologist diagnosis and discretion. There was a significant association between GRS-ALL and severity (SE = 0.0152, *p* = 0.045, OR 1.02 [1.00-1.06]) and between GRS-HLA and severity (SE = 0.027, *p* = 1.64 × 10^−3^, OR 1.09 [1.03-1.15]; Figure 3A; Supplementary Table S6). Meanwhile, there was a non-significant association between GRS-noHLA and severity. To ensure these results were not driven by non-plaque forms of psoriasis (e.g., pustular, erythrodermic) being considered more severe, a sensitivity analysis was performed that included only plaque psoriasis (n = 361), which also confirmed the association of GRS-ALL and GRS-HLA with severity while there was no significant association with GRS-noHLA. These results suggest that the HLA SNPs are primarily responsible for psoriasis severity.

### 3.4 Locations ever affected by psoriasis

Linear regression revealed that GRS-ALL correlated positively with the total number of locations ever affected by psoriasis (*p* = 1.25e-10, SE = 0.023, n = 649, OR 1.16 [1.11-1.21]; Supplementary Figure S1), with a stronger positive correlation observed between GRS-HLA and total locations ever affected (coefficient = 0.295, *R* ^2^ = 0.077, *p* = 3.37 × 10^−13^, SE = 0.040, n = 649, OR 1.34 [1.24-1.45]). Meanwhile, there was a nonsignificant weaker positive correlation when using GRS-noHLA.

A logistic regression was also performed to evaluate the effects of GRS on appearance of psoriasis at 30 different locations (Supplementary Table S7). GRS-ALL and GRS-HLA were both significantly associated with a number of individual body locations. With respect to the incidence of psoriasis at specific locations, only GRS-noHLA had a significant effect on the appearance of psoriasis in the genital area, which was not observed in GRS-ALL and GRS-HLA. These results indicate that patients with higher GRS have more total body locations ever noted with psoriasis, many of which are driven by HLA SNPs; however, the genital area was strongly associated with non-HLA SNPs.

Intrigued by this novel association of genital psoriasis with non-HLA SNPs, we examined this in an independent UCSF cohort of 345 European psoriasis patients of which 32% have genital psoriasis (110 genital, 235 non-genital). In this cohort, genital psoriasis was also found to be strongly associated with GRS-noHLA and GRS-ALL (*p* < 0.05) while it was not associated with GRS-HLA (Supplementary Table S7). This independent dataset provides additional evidence for the association between genital psoriasis and non-HLA SNPs.

To explore the biological basis for the association between non-HLA SNPs and genital psoriasis, an association test between the 88 loci and genital psoriasis was performed. Of these, five SNPs and four SNPs were found to be significant in the Newfoundland and UCSF cohort, respectively, but were found to be insignificant after false discovery rate (FDR) adjustment (Supplementary Table S4). A principal component analysis was also performed to identify which non-HLA SNPs contribute the greatest discriminatory power for genital *versus* non-genital psoriasis (Supplementary Figure S2). SNPs identified as contributing to genital psoriasis included those near *IFIH1* (rs3747517), *UBE2L3* (rs2256609), rs12651787, and rs100040411. SNPs identified as contributing to non-genital psoriasis included *IFIH1* (rs2111485), *ERAP1* (rs39841), *DDX58* (rs11795343), rs2057338, and rs8128234.

### 3.5 First location affected by psoriasis

A logistic regression revealed that a high GRS-ALL was significantly associated with the initial presentation of psoriasis at the elbow (*p* = 0.005) and knee (*p* = 0.025) but not with the initial presentation at the other 18 body locations (Supplementary Table S8). This association was more significant when using GRS-HLA (*p* = 1.10 × 10^−5^ and *p* = 1.23 × 10^−3^ for elbow and knee, respectively). There were no significant effects of GRS-noHLA on the first location psoriasis presented.

**FIGURE 4:**
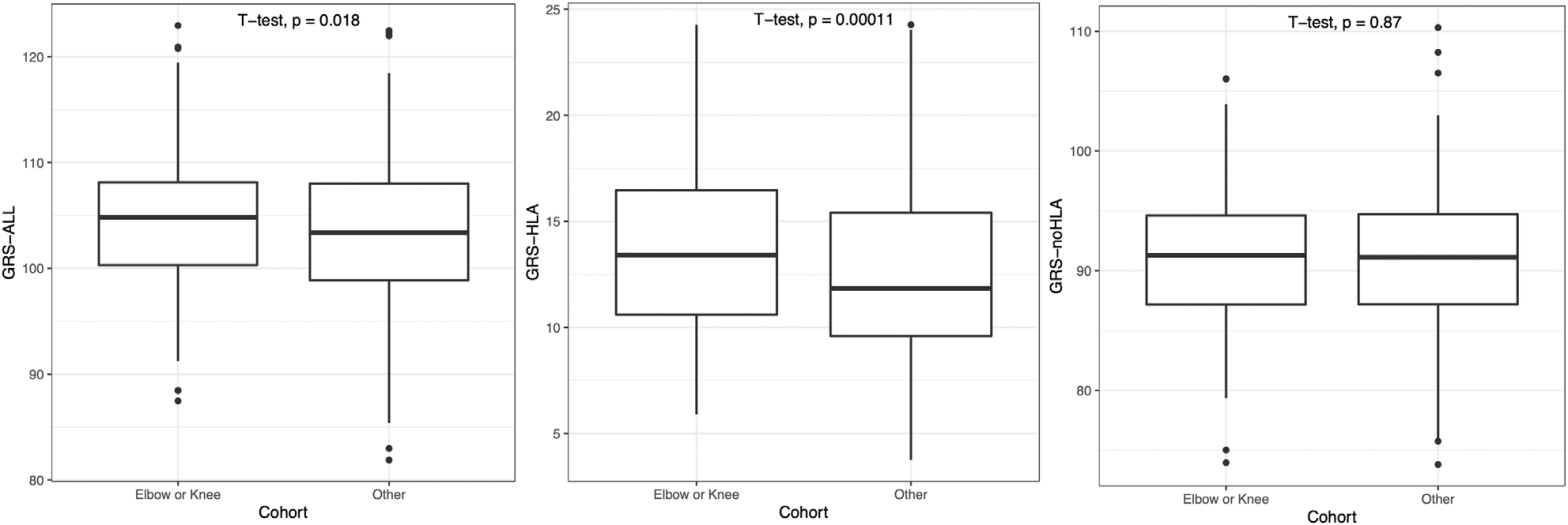
Patients presenting with psoriasis at elbow or knee at first occurrence had significantly higher GRS-ALL and GRS-HLA. Horizontal lines represent quartiles. Points represent outliers. n = 241 (Elbow or Knee), 408 (Other).

As psoriasis often first presents on the elbow or knee location, patients were then grouped into a combined “Elbow or Knee” *versus* “Other” category of first presentation. Patients with “Elbow or Knee” first presentation had a significantly higher GRS-ALL and GRS-HLA compared to those who had psoriasis present at other locations first (*p* = 0.018 and *p* = 0.00011 respectively; Figure 4). Meanwhile, there was no significant difference in GRS-noHLA between those that presented at the “Elbow or Knee” first and those that presented at other locations first. A logistic regression was also performed to evaluate the effect of GRS on the outcome “Elbow or Knee” or “Other”. GRS-ALL and GRS-HLA were significantly associated with presentation at the “Elbow or Knee” first (*p* = 0.019, OR 0.97 [0.94-0.99] and *p* = 0.0001, OR 0.92 [0.88-0.96] respectively) while GRS-noHLA was not (Table 1). Together, these results suggest that HLA SNPs are driving the first presentation of psoriasis at the elbow or knee.

**TABLE 1:** Summary of Results (- if *p* > 0.05, + if *p* < 0.05, ++ if *p* < 0.01, +++ if *p* < 0.001, ++++ if *p* < 0.0001).

		GRS-ALL (or, 95% CI)	GRS-HLA (or, 95% CI)	GRS-noHLA (OR, 95% CI)
Family Score (n = 495)	All	+ (1.02, 1.00-1.04)	-(1.02, 0.98-1.06)	-(1.02, 1.00-1.05)
Onset (n = 571)	All	+++ (0.71, 0.59-0.85)	++++ (0.46, 0.34-0.63)	-(0.90, 0.74-1.10)
	T1P	+++ (0.82, 0.72-0.92)	+++ (0.69, 0.56-0.86)	-(0.90, 0.78-1.03)
	T2P	-(1.34, 0.92-1.96)	-(0.84, 0.44-1.61)	+ (1.54, 1.02-2.33)
Severity (n = 413)	All	+ (1.02, 1.00-1.06)	++ (1.09, 1.03-1.15)	-(1.00, 0.97-1.04)
	T1P	-(1.03, 0.99-1.06)	++ (1.09, 1.03-1.16)	-(1.00, 0.96-1.04)
	T2P	-(0.97, 0.87-1.07)	-(1.01, 0.85-1.21)	-(0.95, 0.85-1.07)
Total Locations (n = 649)	All	++++ (1.16, 1.11-1.21)	++++ (1.34, 1.24-1.45)	-(1.07, 1.02-1.13)
	T1P	++++ (1.13, 1.08-1.19)	++++ (1.34, 1.23-1.47)	-(1.04, 0.98-1.10)
	T2P	-(1.08, 0.95-1.23)	-(1.13, 0.91-1.40)	-(1.05, 0.91-1.21)
Elbow or Knee (n = 649)	All	+ (0.97, 0.94-0.99)	++++ (0.92, 0.88-0.96)	-(1.00, 0.97-1.03)
Genital Psoriasis (n = 649)	All	-(0.96, 0.93-0.99)	-(0.96, 0.91-1.00)	+ (0.97, 0.94-1.00)

### 3.6 Type 1 vs. type 2 psoriasis

**FIGURE 5:**
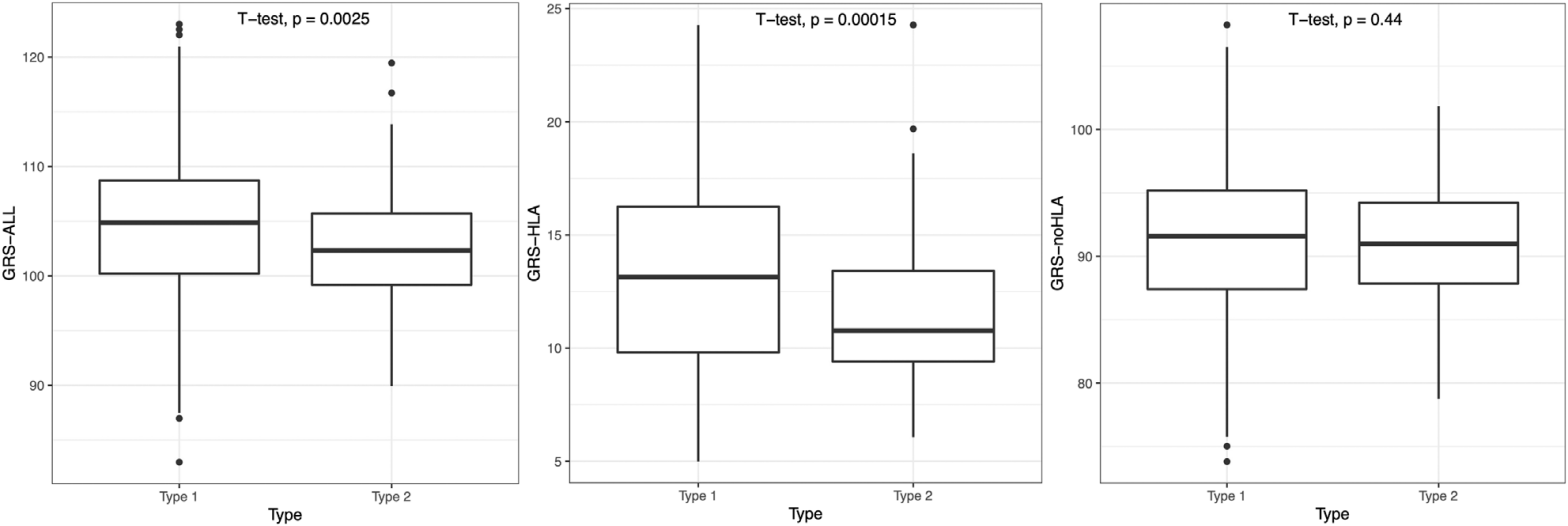
T1P patients had significantly higher GRS-ALL and GRS-HLA. Horizontal lines represent quartiles. Points represent outliers. n = 491 (T1P), 80 (T2P).

The psoriasis subjects were split into an early-onset (age of onset <40) T1P and a late-onset (age of onset **≥** 40) T2P group, and T1P had significantly higher GRS than T2P when using GRS-ALL and GRS-HLA (*p* = 0.0025 and *p* = 0.00015 respectively; Figure 5). A linear regression analysis was performed within each onset group. In T1P, there was a significant negative association between GRS-ALL (coefficient = −0.203, *R* ^2^ = 0.0206, *p* = 0.00084, SE = 0.06, n = 487, OR 0.82 [0.59-0.85]). This was repeated with GRS-HLA which yielded a stronger and more significant association (coefficient = −0.367, R2 = 0.0216, *p* = 0.00067, SE = 0.11, n = 487, OR 0.69 [0.56-0.86]). When this was repeated with GRS-noHLA, the trend was weak and the association was nonsignificant (coefficient = −0.106, *R* ^2^ = 0.0048, *p* = 0.123, SE = 0.07, n = 487, OR 0.90 [0.78-1.03]). In T2P, there was a nonsignificant negative association between GRS-ALL and GRS-HLA with onset; however, there was a significant positive correlation between GRS-noHLA and onset (coefficient = 0.434, *R* ^2^ = 0.041, *p* = 0.039, SE = 0.21, n = 79). These findings suggest an association between non-HLA SNPs and age of onset in the late-onset psoriasis group.

Within the T1P and T2P onset groups, the association between GRS and severity was also analyzed. An ordinal logistic regression was performed, and the only significant association was found in T1P using GRS-HLA (SE = 0.031, *p* = 0.049, OR 1.09 [1.03-1.16]). When comparing the GRS-HLA between the severity levels with a Kruskal–Wallis test followed by a pairwise Wilcoxon rank sum test, there was a significantly higher GRS in the moderate compared to the mild group within T1P (*p* = 0.001, Figure 3B). There was no clear trend and a nonsignificant association in T1P using GRS-ALL and GRS-noHLA an in T2P using all three GRS.

The association between GRS and total number of psoriasis locations was also analyzed within the onset groups. A linear regression analysis revealed a significant association in T1P using GRS-ALL (*p* = 1.40 × 10^−6^, OR 1.13 [1.08-1.19]; Supplementary Figure S1A). This association was stronger and more significant using GRS-HLA (coefficient = 0.124, R2 = 0.045, *p* = 1.401 × 10^−6^, SE = 0.025, n = 487, OR 1.34 [1.23-1.47]; Supplementary Figure S1B) while it was a nonsignificant positive association using GRS-noHLA (Supplementary Figure S1C). The trend was similar but nonsignificant in T2P for all three GRS. Overall, these results indicate that GRS is inversely related with age of onset in the T1P group while not significantly related to age of onset in the T2P group. The GRS was positively associated with psoriasis severity and total locations ever affected, specifically driven by the HLA SNPs within the early onset T1P group.

## 4 Discussion

In this study, we calculated the GRS-ALL, GRS-HLA, and GRS-noHLA and evaluated their correlation to psoriasis clinical features. Of note, among all psoriasis subjects, the mean GRS-ALL was 102.8, mean GRS-HLA was 12.1, and mean GRS-noHLA was 90.6. The fact that GRS-HLA is not the major quantitative contributor to GRS-ALL indicates that associations found significant for GRS-HLA but not for GRS-noHLA were not simply driven by statistical power.

Our data show that patients with a higher GRS-ALL had a higher family score; however, this association is nonsignificant when the GRS was split into GRS-HLA and GRS-noHLA suggesting that all SNPs regardless of loci cumulatively contribute to the family history. This is an interesting finding due to prior research indicating a strong association of family history with *HLA-C06:02* (Schmitt-Egenolf et al., 1993; Schmitt-Egenolf et al., 1996; Gudjonsson et al., 2002). We also found that patients with higher GRS-ALL had an earlier age of onset, and this association was found to be stronger and more significant with the GRS-HLA. This agrees with prior research showing that *HLA-C06:02* -positive patients show an earlier disease onset (Schmitt-Egenolf et al., 1993; Schmitt-Egenolf et al., 1996; Enerback et al., 1997; Gudjonsson et al., 2002). Our data also showed a significant association of GRS-ALL with psoriasis severity level, categorized as mild, moderate, and severe. This association was stronger when using GRS-HLA, suggesting that HLA SNPs could be driving the severity level. Prior studies showed an increasing severity in *HLA-Cw1*-and *HLA-Cw12*-positive patients (Onsun et al., 2019; Huang and Tsai, 2021). Interestingly, the moderate group showed a higher average GRS than the severe group which could be due to a variety of reasons. Besides random noise from sampling, this could in theory be due to specific rare psoriasis mutations, not captured by the common variants on SNP arrays, being responsible for the more severe cases of psoriasis.

We also explored the effects of GRS on the body locations ever affected and first affected by psoriasis. Our data showed that patients with a higher GRS-ALL had higher total locations affected by psoriasis. This association was more significant using GRS-HLA, suggesting that HLA SNPs could be driving this. GRS-HLA had a significant effect on the appearance of psoriasis on the hand, toe, nail, face, knee, back, chest, trunk which was unique to GRS-HLA. A prior study showed that HLA-C*06:02-positive patients had more extensive plaques on their arms, legs, and trunk (Gudjonsson et al., 2002). Interestingly, GRS-noHLA had a unique significant effect on the appearance of psoriasis in the genital area. While there has not yet been significant investigation into the pathology of genital or inverse psoriasis, it is known to display features atypical for plaque psoriasis (Knabel and Mudaliar, 2022). There has also been recent identification of rare gene variants in inverse psoriasis patients that have not previously been reported for psoriasis (Goblos et al., 2021).

In our follow-up analysis to explore the association between non-HLA SNPs and genital psoriasis, *IFIH1* (rs3747517) and *UBE2L3* (rs2256609) were found to contribute to genital psoriasis. Meanwhile, *IFIH1* (rs2111485) and *ERAP1* (rs39841) were found to contribute to non-genital psoriasis. Interestingly, a prior study showed that *IFIH1* is important for immune responses to *Candida* fungal infections (Jaeger et al., 2015). In addition, prior studies have associated *IFIH1* (rs3747517) and *UBE2L3* with an inability to clear hepatitis viruses, leading to chronic infection and increased inflammatory states (Liu et al., 2018; Zhu et al., 2019; Yao et al., 2021). *IFIH1* encodes a retinoic acid-inducible gene I (RIG-I)-like receptor that can sense viral RNA in order to establish a proper antiviral host response. UBE2L3 is an E2 ubiquitin conjugating enzyme that aids in regulation of many signaling pathways including NF-κB. *IFIH1* (rs2111485) and *ERAP1,* on the other hand, have previously been associated with spontaneous clearance of hepatitis virus and more efficient antigen processing during COVID-19 infection, respectively, allowing for faster resolution of inflammation (Jiang et al., 2019; D'Amico et al., 2021; Yao et al., 2021). ERAP1 is a key component of MHC class I antigen processing and presentation. Given the diverse and abundant microbiota in the genital area, we hypothesize that inflammation mediated by innate anti-fungal or anti-viral mechanisms could be correlated with the development of genital psoriasis.

Our data also showed that GRS-ALL was significantly associated with the initial presentation of psoriasis at the elbow and knee. This association became more significant when using GRS-HLA, suggesting that HLA variants could be driving this initial presentation of psoriasis at the elbow and knee. It is commonly observed that psoriasis usually first affects the elbows and knees; however, to date, there have been no studies correlating this presentation with genetic susceptibility loci partitioned between the HLA and non-HLA regions as we have done here.

Our study included an in-depth analysis of the effect of GRS within psoriasis types (early-onset T1P vs late-onset T2P). We found that the T1P group had a significantly higher GRS-ALL and GRS-HLA compared to the T2P group. We also found that T1P patients with a higher GRS-ALL and GRS-HLA had an earlier age of onset. Meanwhile, this association was nonsignificant in the T2P patients. Our data also showed that the GRS-HLA was significantly associated with severity in the T1P patients. Additionally, a higher GRS-ALL and GRS-HLA were significantly associated with more locations ever afflicted by psoriasis in the T1P patients. These findings together suggest that the GRS, driven by HLA variants, affect a number of clinical features in T1P patients and not in T2P patients. This agrees with previous studies showing that T1P is associated with HLA-C*06:02 while other mutations are associated with T2P (Schmitt-Egenolf et al., 1993; Hebert et al., 2015; Kepiro et al., 2021). However, extensive research on the direct effects of HLA variants on T1P subphenotypes were not reported as done here.

It is important to note that this study focused on drawing direct correlations between clinical data and HLA SNPs and non-HLA SNPs. A separate study (manuscript in preparation) discusses the power of this GRS to distinguish phenotype association across multiple cohorts. Additionally, we chose to use the most robust SNPs from a prior meta-analysis to reduce noise from less informative SNPs; however, including additional SNPs could be useful for investigating more subtle genetic associations. Other GRS tools such as PRSice2 and PRS-CS can be utilized to optimize this SNP selection. While we have presented a hypothesis on why the non-HLA SNPs that had higher contribution to genital psoriasis, further studies are needed to understand what specific pathways could be driving this. With a larger dataset, additional partitions can be made to investigate the role of specific immune pathways.

In summary, we examined the relationship between GRS-ALL, GRS-HLA, and GRS-noHLA with multiple psoriasis clinical features within a Newfoundland cohort. We found that the GRS-ALL was significantly associated with positive family history while both GRS-ALL and GRS-HLA were significantly associated with early age of onset, severity, first presentation on elbow and knee, and total locations affected, especially in the early onset T1P patients. Our findings also reveal that non-HLA SNPs influence age of onset in late-onset psoriasis as well as the presence of genital psoriasis. While some of these trends support current knowledge regarding the role of HLA in psoriasis, to our knowledge, there have not yet been studies methodically looking at non-HLA SNPs with clinical data. Nor have there been studies looking at specific body locations and their association with HLA and non-HLA SNPs. Our findings advance our knowledge of how different psoriasis susceptibility variants influence the clinical expression of disease.

## Data Availability

The original contributions presented in the study are publicly available. This data can be found here: https://doi.org/10.6084/m9.figshare.21970847.
