# Supplementary material for: A partitioned 88-loci psoriasis genetic risk score reveals HLA and non-HLA contributions to clinical phenotypes in a Newfoundland psoriasis cohort
Source: Front Genet. 2023 May 31;14:1141010. doi: 10.3389/fgene.2023.1141010 (PMC10265743; doi:10.3389/fgene.2023.1141010)
Supplement: Supplementary file 3 [file Image4.PDF]

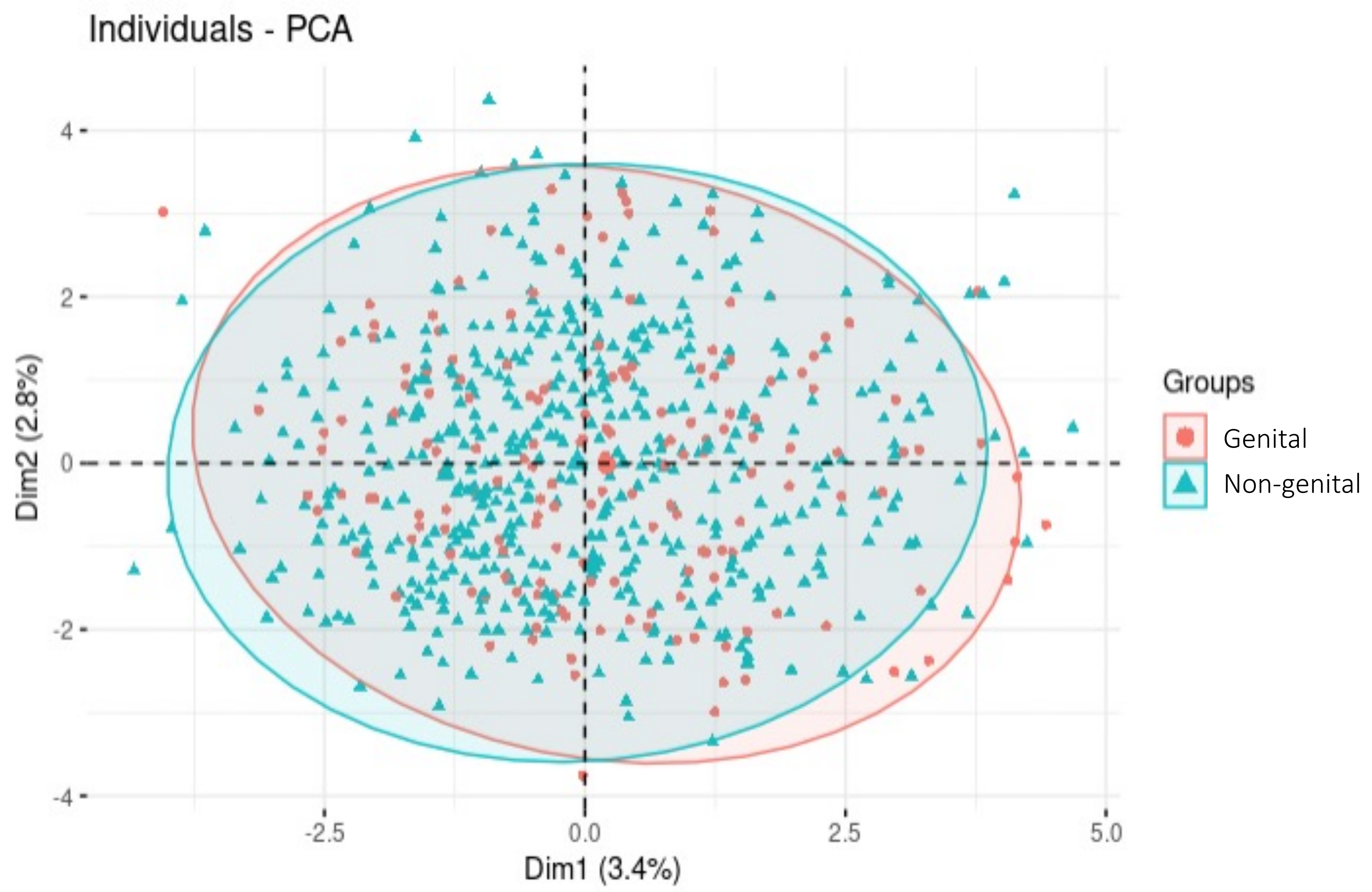

RSID	PC Loading	Gene
rs3747517	0.149624	*IFIH1*
rs7637230	0.10123183	*LINC02085*
rs7705093	0.11659824	*LNPEP*
rs112377695	0.24470113	*IL12B*
rs12188300	0.34221652	-
rs10040411	0.32828416	-
rs12651787	0.34182201	-
rs3759094	0.11850321	*PA2G4*
rs2256609	0.13031789	*UBE2L3*
rs4712528	0.13423121	*CDKAL1*
rs2111485	-0.144614	*IFIH1*
rs39841	-0.1285238	*ERAP1*
rs8177833	-0.0852388	*TNIP1*
rs918520	-0.4772435	-
rs12188300	-0.2237127	-
rs11795343	-0.0953956	*DDX58*
rs3802826	-0.0922724	*ETS1*
rs2057338	-0.0881889	-
rs2199036	-0.1094573	*STX1B*
rs8128234	-0.0700332	-
