# Supplementary figures and images for: A partitioned 88-loci psoriasis genetic risk score reveals HLA and non-HLA contributions to clinical phenotypes in a Newfoundland psoriasis cohort
Source: Front Genet. 2023 May 31;14:1141010. doi: 10.3389/fgene.2023.1141010 (PMC10265743; doi:10.3389/fgene.2023.1141010)

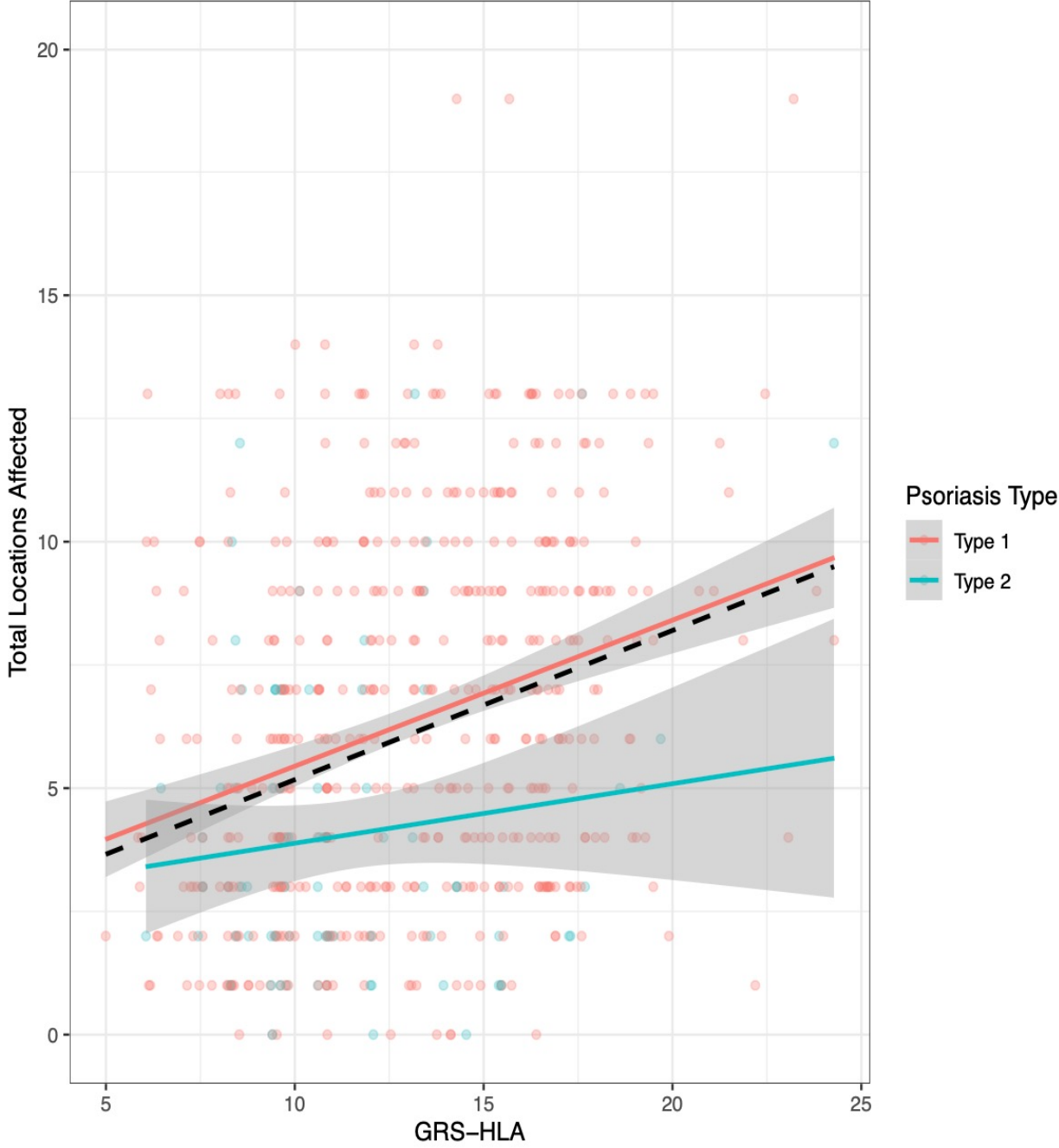

Supplement: Supplementary file 4 [file Image2.PDF]

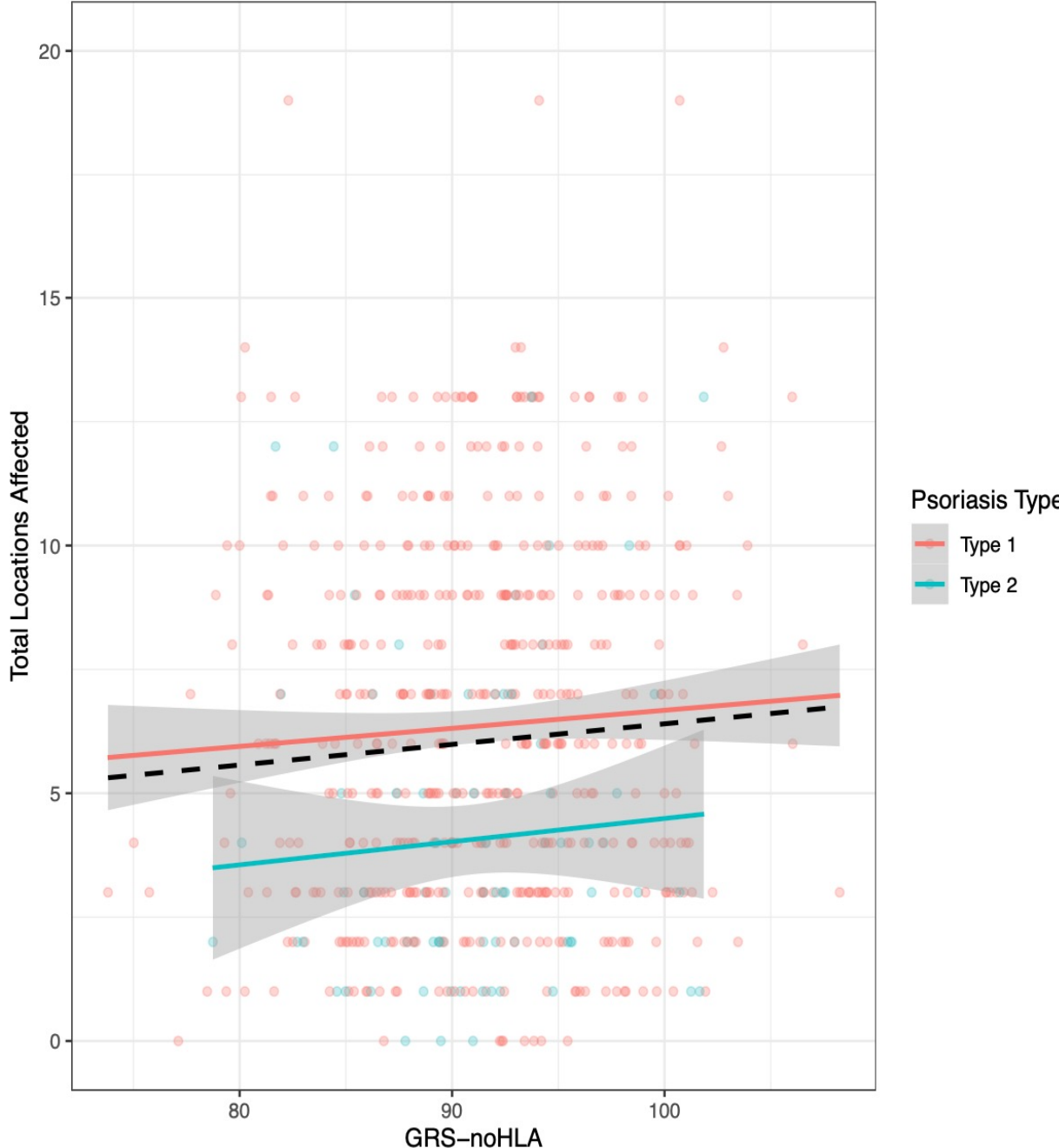

Supplement: Supplementary file 5 [file Image3.PDF]

Total Locations Affected

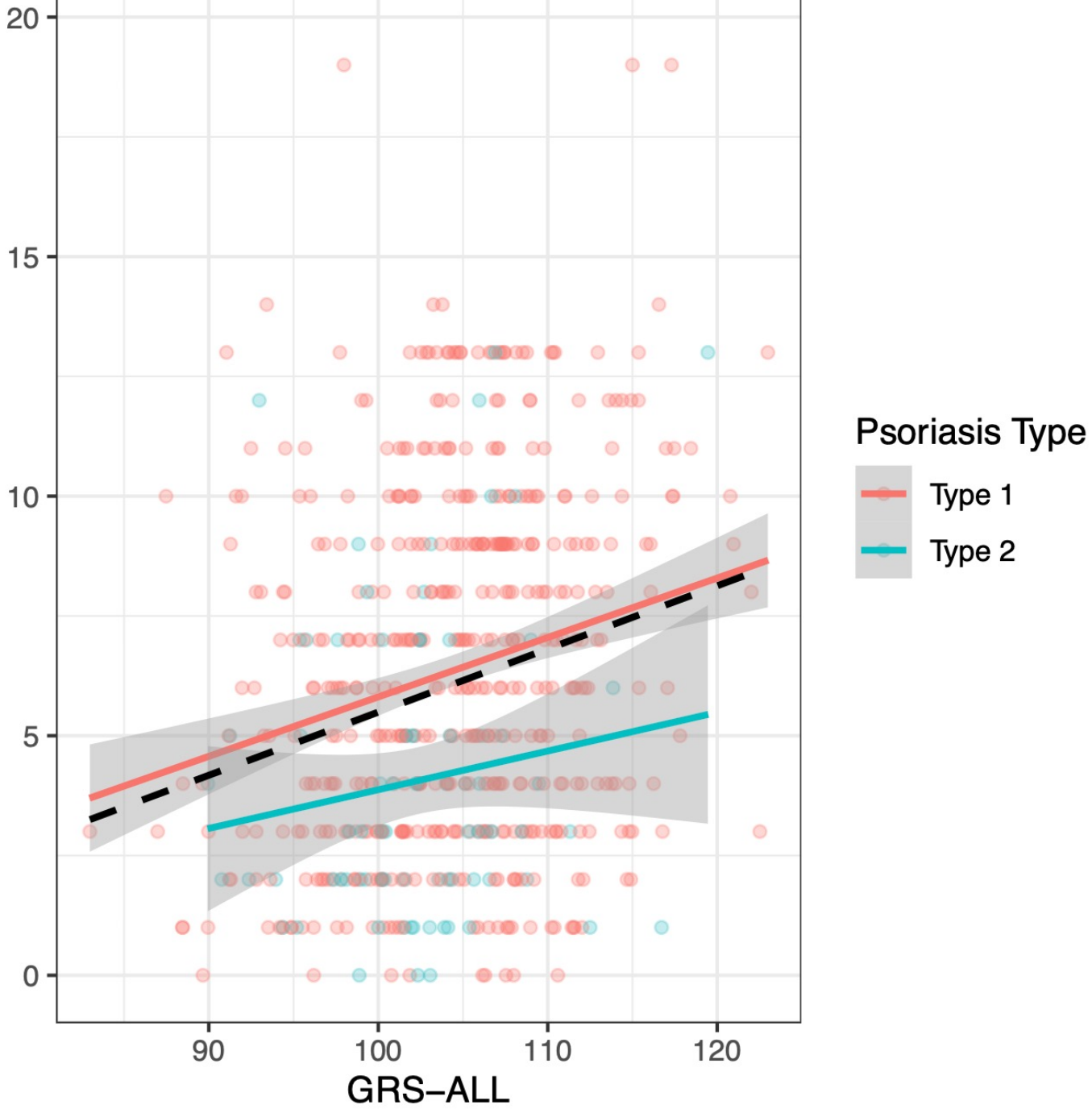

Supplement: Supplementary file 11 [file Image1.PDF]
