# Supplementary material for: A partitioned 88-loci psoriasis genetic risk score reveals HLA and non-HLA contributions to clinical phenotypes in a Newfoundland psoriasis cohort
Source: Front Genet. 2023 May 31;14:1141010. doi: 10.3389/fgene.2023.1141010 (PMC10265743; doi:10.3389/fgene.2023.1141010)
Supplement: Supplementary file 10 [file DataSheet1.DOCX]

Supplementary Material

# Supplementary Figures

**Supplementary Figure 1. Correlation between GRS and total locations ever affected, stratified by psoriasis type. A) GRS-ALL.** In all cases (dashed line), coefficient = 0.149, R^2^ = 0.061, p = 1.25e-10, SE = 0.023, n = 649). In the T1P group (pink), coefficient = 0.124, R^2^ = 0.045, p = 1.40e-6, SE = 0.023, n = 487. In the T2P group (blue), coefficient = 0.081, R^2^ = 0.0077, p = 0.21, SE = 0.064, n = 79. **B) GRS-HLA.** In all cases (dashed line), coefficient = 0.295, R2 = 0.077, p = 3.37e-13, SE = 0.04, n = 649). In the T1P group (pink), coefficient = 0.124, R2 = 0.045, p = 1.401e-6, SE = 0.025, n = 487). In the T2P group (blue), coefficient = 0.081, R2 =0.0077, p =0.21, SE = 0.06, n = 79. **C) GRS-noHLA.** In all cases (dashed line), coefficient = 0.069, R2 = 0.009, p = 0.01, SE = 0.027, n = 649). In the T1P group (pink), coefficient = 0.298, R2 = 0.084, p = 3.94e-11, SE = 0.043, n = 487). In the T2P group (blue), coefficient = 0.121, R2 =0.003, p = 0.266, SE = 0.11, n = 79.

**Supplementary Figure 2. Principal component analysis (PCA) of genital psoriasis.** The first two axes (Dim1 and Dim2) of the PCA are shown with samples grouped as having genital (pink) or non-genital (blue) psoriasis. For Dim1, the top ten non-HLA SNPs with the loadings of the greatest magnitude are listed with their corresponding genes per group.

# Supplementary Tables

**Supplementary Table 1.** Patient questionnaire and data fields.

**Supplementary Table 2.** SNPs used to calculate the GRS.

**Supplementary Table 3.** Imputed SNPs.

**Supplementary Table 4.** Association testing between the 88 loci and genital psoriasis.

**Supplementary Table 5.** Average age of onset per GRS-ALL quartile.

| **GRS-ALL Quartile** | **Average Age of Onset** | **SD** | **N** | **SE** |
| --- | --- | --- | --- | --- |
| Q1 | 27.28 | 13.76 | 164 | 1.07 |
| Q2 | 26.54 | 15.94 | 164 | 1.25 |
| Q3 | 23.21 | 12.89 | 163 | 1.01 |
| Q4 | 20.63 | 12.43 | 163 | 0.97 |

**Supplementary Table 6.** Ordinal regression statistics for effects of GRS on severity.

|  | **Type** | **N** | **Coefficient** | **R2** | **p** |
| --- | --- | --- | --- | --- | --- |
| GRS-ALL | - | 649 | 0.149 | 0.061 | 1.25e-10 |
| GRS-HLA | - | 649 | 0.295 | 0.077 | 3.37e-13 |
| GRS-noHLA | - | 649 | 0.069 | 0.009 | 0.010 |
| GRS-ALL | Type 1 | 487 | 0.124 | 0.045 | 1.40e-06 |
|  | Type 2 | 79 | 0.081 | 0.008 | 0.21 |
| GRS-HLA | Type 1 | 487 | 0.296 | 0.084 | 3.94e-11 |
|  | Type 2 | 79 | 0.121 | 0.003 | 0.27 |
| GRS-noHLA | Type 1 | 487 | 0.036 | 0.001 | 0.21 |
|  | Type 2 | 79 | 0.047 | -0.007 | 0.512 |

**Supplementary Table 7.** Logistic regression statistics for effects of GRS on appearance of psoriasis at 30 different body locations.

**Supplementary Table 8.** Logistic regression statistics for effects of GRS on first appearance of psoriasis at 30 different body locations.
